# Lineage tracing: technology tool for exploring the development, regeneration, and disease of the digestive system

**DOI:** 10.1186/s13287-020-01941-y

**Published:** 2020-10-15

**Authors:** Yue Zhang, Fanhong Zeng, Xu Han, Jun Weng, Yi Gao

**Affiliations:** 1 Department of Hepatobiliary Surgery II, Guangdong Provincial Research Center for Artificial Organ and Tissue Engineering, Guangzhou Clinical Research and Transformation Center for Artificial Liver, Institute of Regenerative Medicine, Zhujiang Hospital, Southern Medical University, Guangzhou, Guangdong Province China; 2 State Key Laboratory of Organ Failure Research, Southern Medical University, Guangzhou, China

**Keywords:** Lineage tracing, Gene targeting, Liver regeneration, Hepatic progenitor cells, Liver disease, The hepatitis B virus, Intrahepatic cholangiocarcinoma, Hepatocellular carcinoma, Gastrointestinal disease, Medical application

## Abstract

Lineage tracing is the most widely used technique to track the migration, proliferation, and differentiation of specific cells in vivo. The currently available gene-targeting technologies have been developing for decades to study organogenesis, tissue injury repairing, and tumor progression by tracing the fates of individual cells. Recently, lineage tracing has expanded the platforms available for disease model establishment, drug screening, cell plasticity research, and personalized medicine development in a molecular and cellular biology perspective. Lineage tracing provides new views for exploring digestive organ development and regeneration and techniques for digestive disease causes and progression. This review focuses on the lineage tracing technology and its application in digestive diseases.

## Introduction

During embryonic development, every single cell assumes different roles of movement, migration, and differentiation to satisfy special organ or system physiological needs. Therefore, tracing the fates of specific cells provides important understandings for monitoring organogenesis, physiological, and pathological processes [1, 2]. The principle of lineage tracing is to track and observe physiological and pathological changes in single-cell level by specific exogenous and endogenous cell markers.

Conklin found that the early mitotic bulb coloration of sea squirt embryos differed in the early twentieth century which was considered as the very beginning of lineage tracing [3]. Later, Cheng and Leblond used iron hematoxylin and ^3^H-thymidine treatment to stain mature enteroendocrine cells to trace differentiation pattern of enteroendocrine cells by electron microscopy; however, inaccuracy of the pigmentation differences limited its further application [4]. Subsequently, Mio succeed observing embryonic development used time-lapse cinematography to monitor the living embryos development which were widely used to study morphogenetic and development changes during the embryonic dynamics [5, 6]. Later, researchers tried to physically inject a variety of dyes and probes into cells and observe the differences in expression between labeled cells and unlabeled cells [6]. However, exogenous markers gradually become diluted as the cell proliferate and cannot effectively label progeny cells [7]. With the help of mature genetic engineering technology, stable gene-targeting technologies have been applied for cell lineage tracing, which overcome traditional lineage disadvantage of the short-term and low specificity [8]. More importantly, the tracers could be continuously observed in proliferation cells.

**Fig. 1:**
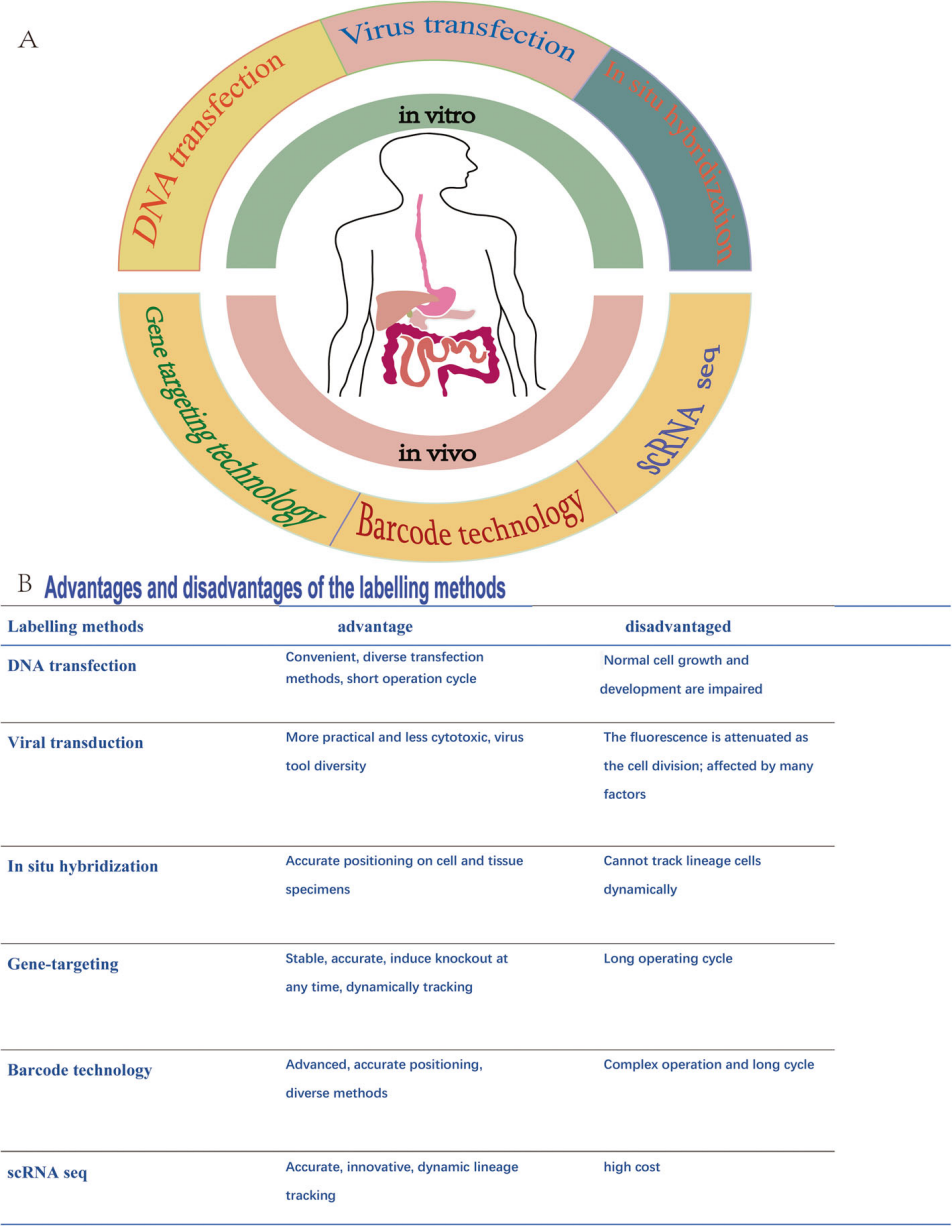
**a** The evolution of lineage tracing technology. In vitro methods: DNA transfection, viral transduction, and in situ hybridization; in vivo method: gene targeting technology, barcode technology, and scRNA seq. **b** Advantages and disadvantages of the labeling methods

Gene-targeting technology utilizes homologous recombination such as the Cre-loxp and Dre-rox systems to control the expression of Cre in specific cells to achieve knockout or modification of target gene. Lineage tracing gene targeting owns the advantage of improved accuracy and integrity which reduced the experimental animal number. It can also be used in the single object at different time points to monitoring different dynamic changes in real time. To date, gene targeting has been widely used in studies of organogenesis, disease models, and susceptibility [9–11]. Several advanced lineage tracing approaches, such as DNA barcode technology and single-cell RNA sequencing (scRNA-seq), have recently emerged to monitor organ formation and tissue damage and regeneration [12–14]. In this review, we focus on the evolution of lineage tracing technology and review its applications in the digestive system (Fig. 1).

## Main text

### Labeling method

#### DNA transfection and viral transduction

In principle, DNA transfection and viral transduction mediate the active or passive introduction of foreign DNA fragments into eukaryotic cells to generate cells with new phenotypes. The difference between these two methods lies in the source of the foreign DNA virus vector DNA and host DNA, respectively. With continued advances in molecular biology research, transfection has become a routine approach in biological experiments, such as those studying the gene function and the regulation of gene expression.

**Fig. 2:**
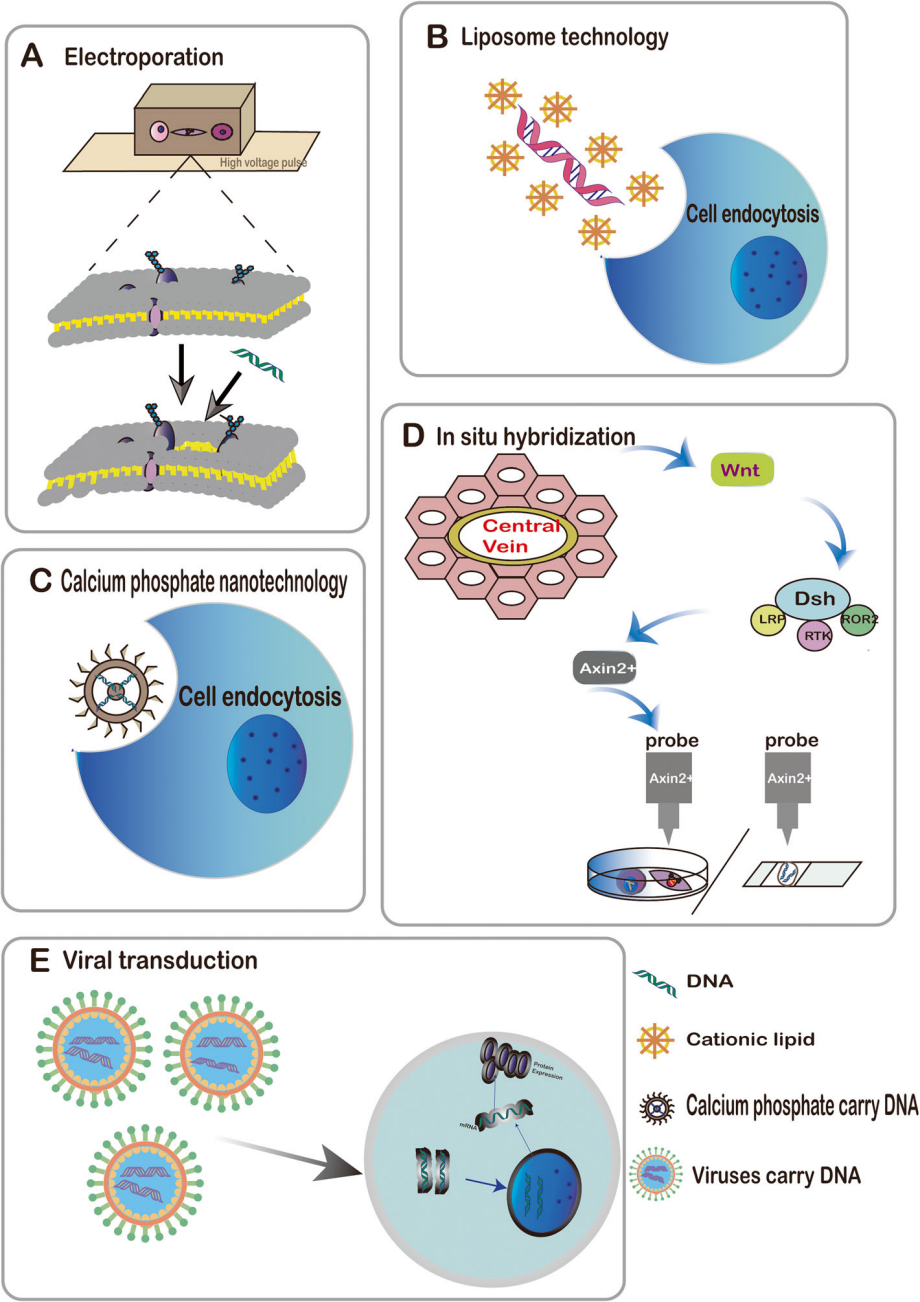
In vitro methods. **a**–**c** DNA transfection; **a** electroporation, **b** liposome technology, **c** calcium phosphate nanotechnology, **d** in situ hybridization, and **e** viral transduction

In 1982, Neumann et al. developed a DNA transfer model by electroporation, since nucleic acids themselves do not actively penetrate the cell membrane, Eid and Sollner efficiently introduced foreign DNA into trypanosomal cells and was transcribed into RNA in a targeted manner (Fig. 2a) [15, 16]. This technology can be regulated by the intensity of the electric shock, and DNA solubility was used to analyze specific expression. Moreover, a variety of cell transfection methods have emerged, such as calcium phosphate nanotechnology and liposome technology (Fig. 2b, c) [17, 18]. However, transfection impairs the normal growth and development of cells and is rarely used in lineage tracing [19, 20].

In contrast, viral transduction is more practical and less cytotoxic than transfection; it uses tools such as adenoviruses, adeno-associated viruses, and lentiviruses to integrate foreign genes into host cells (Fig. 2e). Lentiviral vectors are used mostly for in vitro cell transduction experiments [21]. Adenoviruses and adeno-associated viruses can carry not only the green fluorescent protein (GFP) gene but also other specific genes that allow cells to express specific proteins in vivo [6]. However, the fluorescent labeling method has limitations, such as fluorescence is significantly attenuated during cell division [22]. In contrast to DNA transfection, viral transduction is affected by many factors. In 2019, Hotter et al. found that IFI16 is an antiviral factor, which induces the expression of IFN in CD4+ T cells and macrophages and then inhibits viral transcription by interfering with sp1-dependent gene expression [23]. After viral transduction, the host cell will produce a large number of cytokines, which can directly eliminate the virus or initiate the adaptive immune response of the body. In addition to IFI16 mentioned above, transcription factor IRF3 and its regulatory factor RBCK1 can also regulate the antiviral response of cells, thus affecting the application of virus transduction in lineage tracing [24, 25].

#### In situ hybridization

In situ hybridization refers to a process in which a specific nucleic acid sequence probe is used to hybridize a specimen section sequence accurately and quantitatively. In situ hybridization can be performed on cell or tissue specimens. By in situ hybridization, Zhao et al. found that after liver injury, hepatocytes specifically express the wnt target gene Axin2, and wnt signaling pathway is a key regulator of the fate of stem cells, regulates gene transcription, is involved in multi-organ formation, and also affects the occurrence of tumors [26] (Fig. 2d). Results indicated that Axin2 expressing cells could repair the damaged liver parenchyma [27]. A recent study used Axin2 lineage tracking in transgenic mice and found that liver regeneration is achieved through hepatocyte proliferation rather than putative peripheral stem cells after partial hepatectomy [28]. In summary, in situ hybridization technology was used to show that wnt signaling plays a vital role in liver regeneration throughout the damage repair process.

#### Gene-targeting technology

The combination of embryonic stem cell and homologous recombination technology enables protein-coding genes that carry genetic information to be inherited and stably expressed in organisms [29, 30]. In the 1980s, Thomas and Capecchi used gene-targeting techniques to perform site-directed mutagenesis of endogenous genes in mouse embryonic stem cells [29]. Gene-targeting technology including primarily the FLP/FRT site-specific recombination system from yeast [31, 32], the Cre-loxp recombinase system from *Escherichia coli* phage P1, and the emerging Dre-rox recombination system. The yeast FLP/FRT site-specific recombination system is used in botanical studies, and the Cre-loxp and Dre-rox recombination systems are the most common gene targeting techniques currently used in non-plant organisms.

The Cre-loxp recombinase system can accelerate the genetic modification of experimental animals, effectively identify unique sites in lineage tracing process. Gene targeting is generally divided into two steps. First, the loxp sequence is introduced into an embryonic stem cell genome [33]. Second, the loxp site is specifically recognized and cut by Cre recombinase to achieve genetic modification or mutation of the target gene. Before Cre promoter gene sequence of specific cells can be inserted to conduct the cell lineage accurately. The commonly used hepatocyte-promoter: Alb, the stem cell specific promoter: Lgr5, etc. The Cre-loxp recombinase system was used in ROSA26 mice to generate mT/mG mice, in which cells can be marked with different fluorescence according to their identities, thus greatly improving the resolution of the tracer [34].

Cre-ERT2 mice have also been used in lineage tracing and express a fusion protein of an estrogen receptor (ER) ligand binding region mutant (ERT). Expression or deletion of mutation sites depend on regulation by tamoxifen, whose metabolite 4-OHT (estrogen analog) binds to ERT, inducing Cre-ERT2 enter the nucleus to activate Cre recombinase [35]. In the Cre-ERT2 mouse model, the mutation time can be adjusted via the timing of tamoxifen interference. In 2010, Quante et al. used TFF2-Cre (ERT2) mice for lineage tracing and observed TFF2 mRNA is expressed in T cells under tamoxifen induction, these cells are located in the isthmus of gastric glands. These cells were divided by cell markers into progenitors of mucus neck, parietal, and zymogen-secreting cells in the gastric mucosa [36]. However, in 2012, researchers found that tamoxifen is gastrotoxic administered orally or intraperitoneally. Within 3 days of drug administration, chief cell metaplasia and even apoptosis occurred in 90% gastric parietal cells [37, 38], it may have off-target effect in the process of lineage tracing [39]. Thus, the application of tamoxifen requires continuous optimization in lineage tracing processes, such as the use of tamoxifen metering, management methods. After eliminating these confounding factors, the experimental conclusions can be analyzed accurately.

Cre recombinase may induce gene mutations, and deletions can easily cause abnormal embryonic development, leading to embryonic lethal. In early studies of some oncogenes with the cre-loxp technology, homozygous deletion causes embryonic death under certain circumstances, although the development of heterozygotes was normal [40, 41]. Moreover, some homozygous female transgenic mice have severely impaired uterine development and function, which may lead to infertility [42]. Recently, Álvarez-Aznar et al. used tamoxifen for temporal control of mutations by the CreERT2/loxP system to regulate the time of fetal death and study the effects of lethal genes in later development [43].

**Fig. 3:**
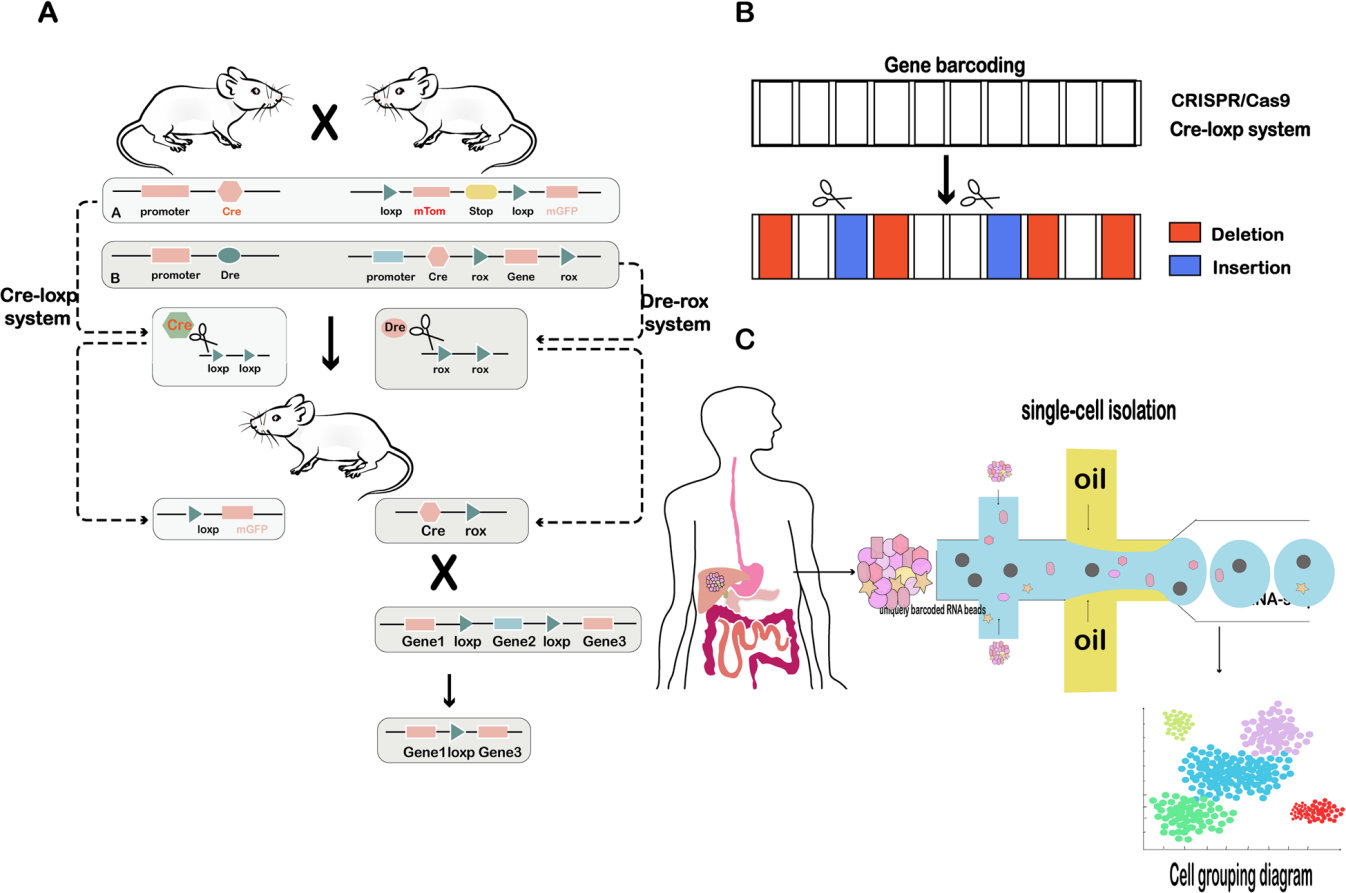
In vivo method: gene targeting technology: **a** Cre-loxp recombinase system, Dre-rox recombinase system; **b** gene barcoding; and **c** single-cell sequencing methods

However, in 2017, Lingjuan et al. described a new lineage tracing system that combines the Dre-rox and cre-loxp recombination systems to improve the accuracy and selectivity of traditional cre-loxp lineage tracing (Fig. 3a). The combination of these two orthogonal recombination systems can effectively and specifically target organs to explore its development and damage repair, even understanding the strong plasticity of progenitor cells in vivo [44, 45]. In addition to the above applications, this dual-enzyme activation lineage tracing approach is a valuable strategy for precisely targeted genetic manipulation in mammals. Researchers have used more stringent hybridization methods to screen cells that express the protein, allowing the most accurate labeling.

#### Barcode technology

Genetic barcode technology has been used for species identification and classification, and it has been advanced substantially by gene editing. McKenna et al. developed the genome editing of synthetic target arrays for lineage tracing (GESTALT) approach to trace cell lineages. During zebrafish development, unmodified CRISPR/Cas9 target sites were edited to combine diverse sequences into multi-allelic barcodes, and single-cell sequencing was performed [12]. Later, on the basis of the traditional Cre-loxP system, the Rodewald laboratory developed a barcode reagent to rearrange or remove specifically labeled DNA fragments, interfering with hematopoietic stem cells in mice. This barcode did not affect the physiological development of the labeled mice and was retained as the hematopoietic stem cells divided and matured [46]. This technology accumulates the combined sequence by deletion and insertion into a compact and informative barcode based on CRISPR/Cas9 and Cre-loxp system (Fig. 3b). It is suitable for any organism and building the construction of a hierarchical development tree based on comprehensive barcodes to track the origin of individual cells. In addition, in 2018, a new barcode technology was reported in the Sleeping Beauty transposon system, which is composed of a transposase and a transposon, it can insert specific DNA sequences into animal genomes to barcode mouse cells. In the corresponding article, the researchers used the transcriptional activator M2/hyperactive Sleeping Beauty/transposon (M2/HSB/Tn) mouse model, random transposon integration was induced by adding doxycycline, and red fluorescent protein was used to label cells to track blood progenitor cells during blood regeneration [13, 47]. Subsequently, a new type of Sleeping Beauty transposon was developed to generate chimeric antigen receptor T cells and applied in cancer immunotherapy [48]. Previously, due to the lack of appropriate tools to study the natural formation of blood cells, possibly highlighting the practicality and innovation of this technology, significant advances have been made in both therapeutics and gene editing, as it improves fidelity and increases the safety of genomic modifications.

#### Single-cell sequencing methods

scRNA-seq has become popular in recent years. This technique can determine the abundance of RNAs with high accuracy and high sensitivity, but it can only capture a static snapshot at a single time point [49]. In 2018, La Manno et al. successfully depicted cell fate in a dynamic manner by using a mathematical model and studied a group of differentiated cells in the mouse Hippo campus which is a key pathway for homeostasis, analyzing the RNA velocity in more than 18,000 cells to determine the future differentiation direction of brain stem cells or intermediate progenitors and predict their ultimate fates [50]. Remarkably, this technique for assessing RNA velocity can facilitate lineage analysis and could help to characterize developmental disorders of the human brain, such as autism and schizophrenia.

In 2018, a major advance was made in single-cell sequencing: it could be used not only to study individual organs but also to track the development of a single cell into a complete organism. Alemany et al. developed a new technology called ScarTrace based on single-cell level. This technology adds tandem copies of a fluorescent protein transgene to label zebrafish cells and can track adult cells in multiple locations, including the kidneys and eyes [14]. However, Plass et al. used the planarian Schmidtea mediterranea, a model organism with almost unlimited regeneration capacity and almost all cells exist in the adult body from pluripotent stem cells to differentiated cells. The Plass group used single-cell transcriptome sequencing to locate all major cell types in a lineage tree at high resolution, with the goal of exploring the cell types that contribute to regeneration [51]. In addition, Professor Vijay G. Sankaran exploited the high mutation rate of mitochondrial DNA (mtDNA) and combined these characteristics with scRNA-seq and single-cell chromatin open sequencing technology (scATAC-seq) [52]. The advanced method uses the characteristics of mtDNA mutational diversity as the endogenous barcode to track genetic mutations at the single-cell level, and it can be effectively applied to dynamically track human cell lineages, thus providing technical support for developmental biology research and disease prediction.

### Application

#### Liver

The liver has powerful and complex regenerative functions [53, 54]. However, the mechanism of liver regeneration is still unclear [55, 56]. In 2015, Wang et al. used Axin2-CreERT2 mice to exhibit fluorescence in response to Wnt signaling pathway activation in the liver, and they found stem cells divide continuously which located around the central vein. If liver stem cells escape Wnt signaling, which begin to differentiate into mature hepatocytes quickly [56]. Prior et al. used lineage tracing technology combined with scRNA-seq to analyze the highly expression of Lgr5 in hepatocytes in the early embryonic development stage, and their cells have bidirectional differentiation potential [57].

According to the time of initiation, liver injury is classified as acute and chronic, and the regeneration pathways differ across different types of injuries [58, 59]. In a recent study, Chen et al. used growth regulators as markers for lineage tracing a group of widely distributed hepatocytes with the ability to proliferate and regenerate [60]. In addition, studies have reported that under the condition of acute liver injury caused by hepatectomy and hyperbilirubinemia, liver regeneration is accomplished through the proliferation of liver cells [55, 61]. Tomonori et al. used four-color fluorescence lineage tracing technology combined with flow cytometric sorting technology to research diploid and polyploid liver cell proliferation after chronic liver damage [62]. During liver damage repair, polyploid hepatocytes can proliferate and some hepatocytes exhibit reduced diploidy. Later, Rodrigo-Torres et al. found that ductular reaction cells express Hnf1β in human liver cirrhosis; thus, they used Hnf1βCreER/R26RYfp/LacZ mice, which carry the yellow fluorescent protein (YFP) gene downstream of Hnf1β-cre, to track HNF1+ biliary duct cells. Acute and chronic liver injury was induced in the mice, and the lineage of HNF1+ cells was traced. This study shows that the contribution of Hnf1β to hepatic progenitor cell (HPC) expansion and hepatocyte production is dependent on liver damage, especially chronic liver damage [63]. Although HPCs are normally static, they are activated in the presence of liver damage and differentiate into hepatocytes and biliary epithelial cells (BECs) to support liver regeneration and maintenance of liver function [64, 65]. In addition, Tae-Young et al. used cre-loxp fluorescent zebrafish to trace the lineage of liver cells and found that after treatment with metronidazole, BECs transdifferentiated and proliferated into hepatocytes, restoring the quality of hepatocytes [66]. Subsequently, Russell et al. used Krt19-Cre mice bred with fluorescent ROSA mice to trace the lineage of BECs. Liver injury was induced by administration of a choline-deficient, ethionine-supplemented (CDE) diet; the mice were then recovered on a normal diet, and BECs were shown to differentiate into hepatocytes to maintain liver function [67]. These results indicate that tissue repair and regeneration functions are not limited to stem cell populations; mature hepatocytes and BECs also exhibit plasticity.

Viral infection can cause chronic liver damage and is the main cause of liver cancer worldwide; hepatitis B virus (HBV) infection is the most common such agent. Lineage tracing technology was used to explore the influence of signaling pathways related to HBV infection on disease progression [68, 69]. For example, Lu et al. used the cre-loxp system to specifically knock out the E3 ubiquitin ligase Mdm2 gene in liver cells, leading to apoptosis and necrosis in a large number of liver cells and found that HPCs are activated to participate in liver regeneration and reconstruction [2]. In the setting of liver damage caused by HBV infection, the mdm2/p53 axis of cell cycle regulation-related genes is altered, suggesting that HBV-induced liver damage is related to the HPC phenotype (IGF+, AFP+, EPCAM+), which may activate HPC proliferation and thereby mediate repair and regeneration [70]. Through transcriptome sequencing of human hepatocellular carcinoma (HCC) samples, six genes—TSC1, TSC2, PABPC3, HIF1α, RB1CC1 (ATG17), and RPS6KA3 (RSK2)—were found to be associated with HBV infection [71]. Huang et al. generated mice with liver-specific knockout of TSC1 (TSC1-knockout mice) to activate the mTOR pathway and found that these mice can spontaneously develop liver cancer via a mechanism related to the intestinal flora [72]. However, studies have also shown that the HBV X protein (HBX) activates Ras, rapidly induces the cytoplasmic Ras-Raf-MAP kinase signaling cascade, promotes cell proliferation, and activates transcription factors, thereby causing liver damage [73, 74]. In addition, studies have shown that the interaction between liver damage caused by HBV infection and the HBX protein in malignant tumors may differ according to cell distribution. Therefore, serum response factor (SRF) controlled by Ras/MAPK signal is used as the target for lineage tracing. Lethal HCC develops in mice with specific expression of SRF-VP16, indicating that the Ras/MAPK signaling pathway is a highly oncogenic pathway in HCC [75]. Lee et al. used lineage tracing technology and found that Smad/TGF-β-related signaling pathways are closely related to HBV-induced liver fibrosis [76]. In summary, the abovementioned lineage tracing technology has revealed that most tumors related to HBV infection exhibit DNA damage and P53 protein ubiquitination affects the cell cycle, induces the Ras-Raf-MAP kinase cascade, affects cell transcription and proliferation, and even induces the development of lethal HCC. Multiple signaling pathways are involved in these processes. Surprisingly, liver damage caused by HBV can induce gene overexpression in HPCs, and amelioration of liver damage associated with HBV infection may involve the regeneration and repair of HPCs.

**Fig. 4:**
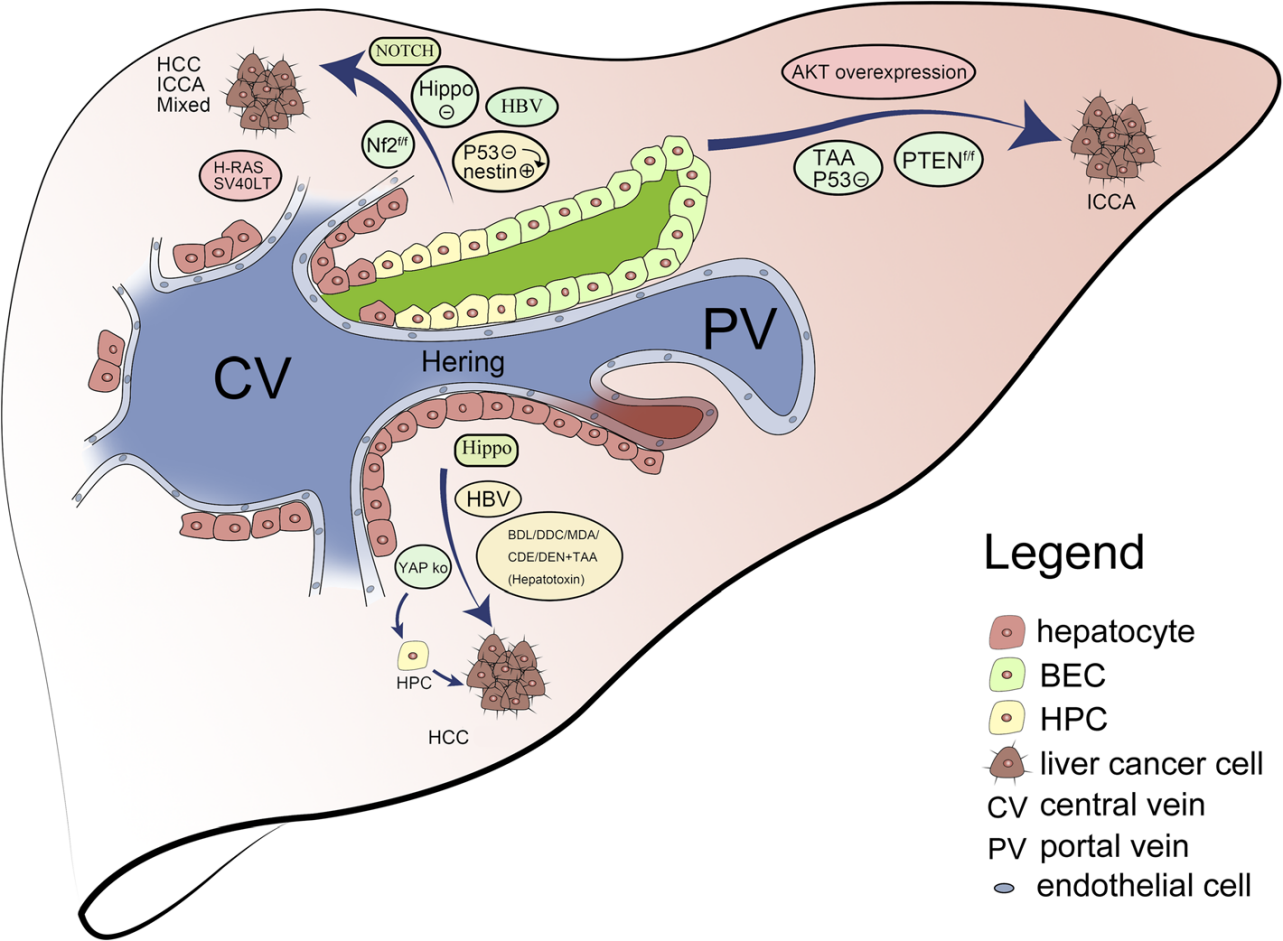
Various factors influence the occurrence of liver cancer. In the case of HBV infection and hepatotoxic injury (DDC/MDA/BDL/CDE/DEN+TAA) and Hippo/YAP, specific knockout induces hepatocyte proliferation into HCC ICCA, inhibiting Hippo NOTCH pathway and deleting Nf2 or P53 to activate nestin protein and HBV infection; HPC proliferation induces HCC, ICCA, and mixed HCC-ICCA; AKT pathway overexpression, PTEN specific knockout, or inhibiting P53 and TAA induce BECs form ICCA

In addition to using lineage tracing to study the repair of liver injury, researchers have also applied lineage tracing to liver cancer research. Primary liver cancers are broadly classified as HCC, intrahepatic cholangiocarcinoma (ICCA), and other rare tumors [77]. HCC is the most common malignant tumor in the liver; ICCA is less common than HCC, accounting for approximately 10–15% of primary malignant tumors in the liver [55, 78, 79]. However, mixed HCC-ICCA is a rare type of tumor. To date, researchers have applied primarily genetically engineered mouse models (GEMMs) and recombinase-mediated genetic lineage tracing technology to establish liver cancer models. Due to the complex mechanisms underlying the occurrence of liver cancer and their homogeneity among individuals, the cellular sources of primary liver cancers of various etiologies have been studied (Fig. 4). It is particularly important to analyze the available data and design new studies from this perspective [80]. For example, during the initiation of HCC, many signaling pathways are inhibited or activated, among which the Hippo pathway is the most classic. It is involved in the regulation of individual development and homeostasis and regulates cell proliferation and apoptosis through a series of related kinase interactions. It is also abnormally expressed in malignant tumors. Li et al. specifically knocked out the homolog Sav1 and the mst1 and mst2 kinases of the Hippo signaling pathway in hepatocytes, thereby inhibiting the Hippo pathway, activating HPCs and inducing liver cancer [81, 82]. YAP is a downstream effector of the Hippo pathway and also a proto-oncogene, which can overexpress to remove the contact inhibition of cells and make cell proliferation uncontrolled. Lineage tracing technology was used to explore the role of YAP in cancer treatment. Small molecules and peptides have been used to inhibit YAP expression in vivo, and studies have shown that when YAP is silenced, tumor cell proliferation is halted and hepatocyte differentiation is activated, leading to tumor regression [83]. In a previous study, Ivan et al. discovered a strange phenomenon: lack of YAP and TAZ in normal hepatocytes surrounding the tumor accelerated tumor growth. Conversely, overexpression of YAP in cells surrounding the tumor contributed to regression of liver cancer [84]; therefore, the survival of liver cancer cells is dependent not on internal YAP/TAZ expression but on YAP/TAZ expression in adjacent liver cells. The above studies used lineage tracing technology to analyze and summarize the two mechanisms of cellular autonomic nerve suppression and non-cellular autonomic tumor suppression and identified additional targets for clinical treatment.

Neurofibromatosis type 2 (Nf2) is a tumor suppressor gene in adult mice. Lee et al. specifically knocked out Nf2 in the liver. Cells of this lineage were traced back to the massive proliferation of HPCs, which eventually led to the development of mixed HCC-ICCA [85]. In addition, Tschaharganeh et al. used the Alfp-Cre p53^fl/fl^ animal model to specifically knock out P53, promote the dedifferentiation of mature hepatocytes into nestin-positive HPCs, and induce the formation of mixed HCC-ICCA by activating WNT and NOTCH pathway which affects multiple processes of normal cell morphogenesis, including the differentiation of pluripotent stem cells, cell apoptosis, and proliferation [86, 87]. In a 2016 study comparing mice with a specific knockout of the autophagy-related gene P62 in liver cells (Sqstm1^Δhep^ mice) and Sqstm1^fl/fl^ control mice, upregulation of P62 during early tumor development induced liver cells to become cancerous [88]. In fact, for the establishment of liver cancer models, the most commonly used drugs are hepatotoxic drugs and diets, such as 3,5-diethoxycarbonyl-1,4-dihydrocollidine (DDC), MDA, CDE, and diethylnitrosamine (DEN); the bile duct ligation (BDL) model and chronic cholestatic injury models are also used. In addition, adeno-associated viruses have been used to carry Cre (AAV-TBG-Cre). Mouse liver cells were specifically labeled by tail vein injection, and lineage tracing showed that the HCC cells in this hepatotoxic injury model were derived from hepatocytes [89].

The worldwide incidence of cholangiocarcinoma is increasing annually [90]. Studies on cholangiocarcinoma have shown that NOTCH signaling plays an important role in this cancer. Steffen Zender used Cre recombinase technology to interfere with NOTCH signaling and found that NOTCH can induce the transformation of dual-potential HPCs into cholangiocarcinoma cells and form cholangiocarcinoma tumors [91]. In addition, PTEN signaling pathway activity exerts an inhibitory effect on ICCA. In a model of Alb-Cre mice crossed with Pten^fl/fl^ mice to generate Pten silencing, aristolochic acid (AA) directly induced liver cancer, including HCC and ICCA [92, 93]. Guest RV labeled the BECs of CK19-CreERTeYFPp53^fl/fl^ mice for pedigree tracking and regularly injected tamoxifen to knock out P53, discovering that chronic BEC damage is the origin of ICCA [94].

Mixed HCC-ICCA is a rare liver tumor that few related studies have addressed. Chiba et al. overexpressed Bmi1 and Wnt/β-catenin in C57 mice and severely immunodeficient mice, which resulted in uncontrolled expansion of HPCs and dysregulation of self-renewal to induce liver cancer exhibiting common histological characteristics of HCC and bile duct cancer [95]. In addition, a study confirmed that the Hippo pathway effector YAP is related to mixed HCC-ICCA. The researchers activated YAP in mice with liver-specific knockout of WW45 and found that the HPCs in these mice swelled and eventually formed liver tumors. Histological analysis showed that these liver tumors exhibited mixed pathological features of HCC and ICCA [85].

The above findings indicate that lineage tracking technology plays an indispensable role in liver cancer research. This technology can be used not only to study the influence of activation or inhibition of certain pathways on liver cancer occurrence and development but also to explore the origins of liver cancer cells. In the future, with logical selection of the activator or inhibitor of each pathway, we can effectively control the occurrence and development of liver tumors.

#### The pancreas

**Fig. 5:**
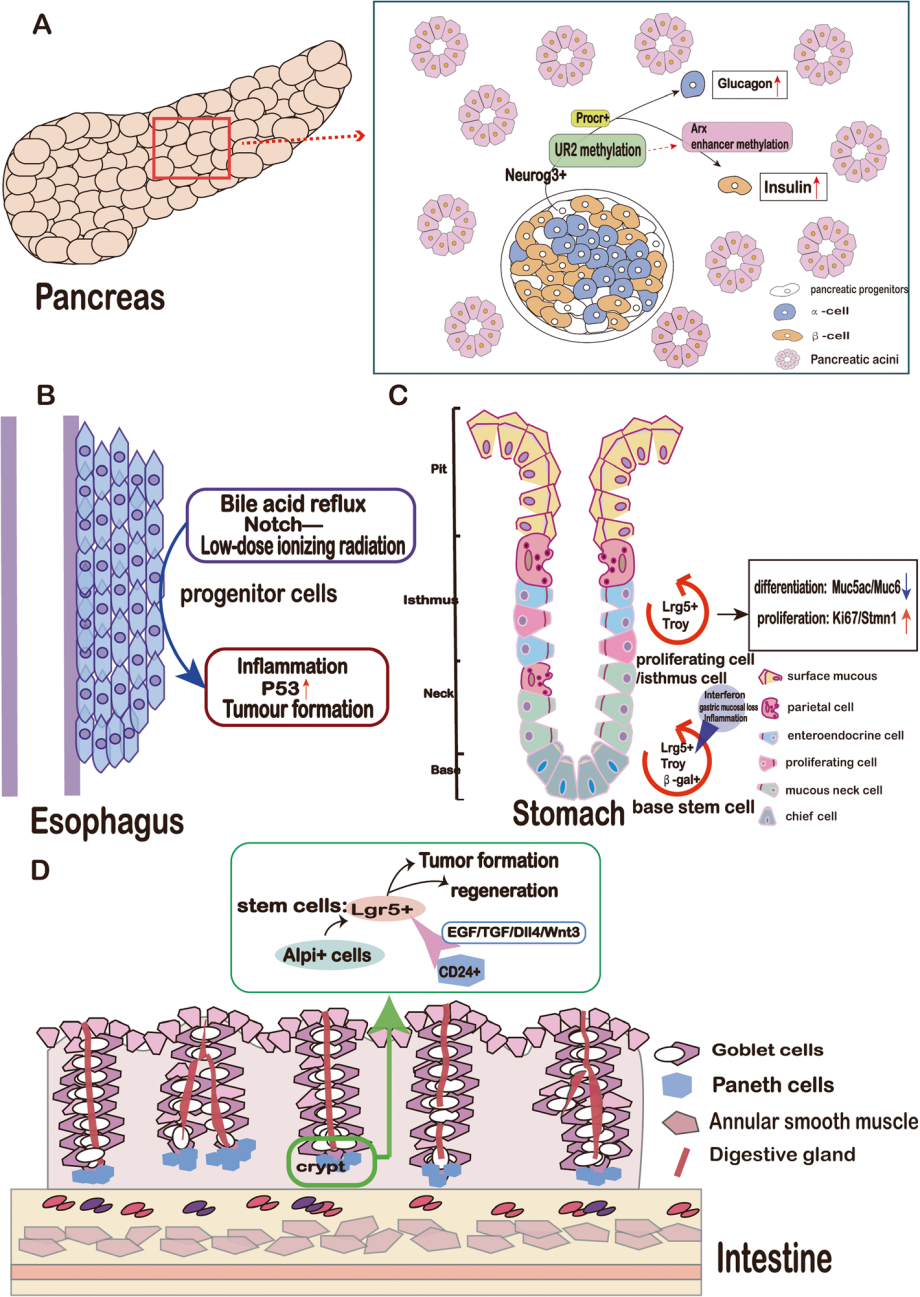
**a** Developmental and regenerative mechanisms of the pancreas. **b** The damage repair process of esophageal progenitor cells. **c** Plasticity potential of gastric base cells and isthmus cells; **d** Lgr5-positive cells with colon crypt structure have stem cell function, and CD24+ Paneth cells can produce growth factors that promote stem cell proliferation

The pancreas and its secretion functions represent an important part of the digestive system. The pancreas is composed of endocrine and exocrine tissues, which regulate glucose homeostasis and produce digestive enzymes, respectively. Since the pancreas, liver, and intestines originate from the embryonic endoderm, many studies have used lineage tracing to track specific markers of endoderm cells to continuously monitor the fates of stem cells in organs such as the pancreas [96–99]. As early as 2002, Kawaguchi et al. carried out lineage tracing of cells expressing Ptf1a, disproving the previous hypothesis that Ptf1a expression is specific to exocrine cells. Lineage analysis showed that Ptf1a is expressed during the early stages of differentiation in progenitor cells of various pancreatic cells [99]. After the development of scRNA-seq, Byrnes et al. used this technique, in situ hybridization and cre-loxp recombinase systems to analyze the interaction between mouse pancreatic epithelial cells and mesenchymal cells and even identified a population of undefined endocrine progenitor cells in the pancreas [100]. Jing et al. used scRNA-seq and gene reporter mice to conduct lineage studies on the pancreas. Their study showed that a population of progenitor cells expressing Neurog3 can differentiate into a variety of endocrine cell subtypes during pancreatic development and that Arx enhancer hyper-methylation contributes to the production of progenitor cells; these discoveries will facilitate mass production of progenitor cells [101]. Next, Daisong et al. used scRNA-seq to discover unidentified protein C receptor-positive (Procr+) cell populations in the pancreas and used lineage analysis to show that these cells can differentiate into endocrine cells; in addition, Procr+ pancreatic organoids transplanted into a mouse model of diabetes regulated blood sugar and secreted insulin, thereby ameliorating diabetes [102] (Fig. 5a). Via a combination of several lineage tracing techniques, these researchers revealed the development and secretion behavior trajectory of the entire pancreas.

#### The esophagus

The main functions of the digestive tract are to process ingested food and to absorb nutrients and water. The digestive system can even communicate with the brain through the nerve and endocrine pathways to maintain energy conservation in the body [103, 104]. Here, we focus on the esophagus, stomach, and intestines. Jiang et al. used lineage tracing methods with fluorescent protein-labeled basal progenitor cells to confirm that bile acid reflux or genetic changes promote the proliferation of these progenitor cells, leading to the development of Barrett’s esophagus [105], a precursor of esophageal cancer. However, common Notch pathway-related genes are expressed in esophageal epithelial cells, and widespread loss of Notch signaling results in inflammation and tumor formation [106–108]. Therefore, Alcolea et al. used mice expressing a dominant negative mutant of mastermind-like 1 (DNM mice), which show inhibition of Notch transcription, and found that all mutation sites were labeled with GFP. Lineage tracing showed that the oncogene P53 not only has a synergistic effect at the cellular level but may also influence the cellular dynamics in early stages of cancer through a field change [109]. Lineage tracing is often used to explore risk factors for cancer. For example, Fernandez-Antoran et al. exposed AhCreERT-R26flEYFP/wt mice to low-dose ionizing radiation, equivalent to the dose received through 3–4 CT scans. Surprisingly, exposure to these seemingly ordinary radiation doses increased the number of P53 mutant cells in the esophagus, and the proliferation rate of these cells suppressed that of healthy cells. However, treatment of the model mice with antioxidants prior to radiation eliminated the P53 mutant cells [110] (Fig. 5b). Research on lineage tracing technology in the esophagus showed that this technology identified not only seemingly minor carcinogens but also an effective solution to prevent the occurrence of esophageal cancer. Thus, lineage tracing will be actively promoted in future clinical applications.

#### The stomach

Lineage tracing technology is also widely used to study the renewal of the gastric epithelium and in research on gastric cancer. As the starting organ in the digestive tract, the stomach controls peristalsis via the surrounding muscles and secretes enzymes and acids from specific regions for food digestion. The upper part of the stomach, the forestomach, is composed of a layer of epithelial cells whose renewal process is unclear. In 2018, Pawel et al. used the Cre-loxp recombinase system to label cell subgroups in various parts of the stomach with Lrig1-GFP and found that in the basal layer of the forestomach and the lower part of the gland, a population of cells expressing Lrig1 can promote long-term maintenance of the gastric epithelium [111]. Later, this group performed in vitro organoid experiments and showed that cells with high Lrig1 expression exhibit an increased spheroidization ability [111]. The literature indicates that in addition to markers of stem cells in the basal area, the identity of stem cells in the isthmus is also controversial. Han et al. used the Confetti mouse multi-color reporter system and scRNA-seq to perform lineage analysis of isthmus cells. This study showed that two different stem cell populations control the gastric glands and that these populations express the cell proliferation markers Stmn1 and Ki67 [112]. The gastrointestinal system is an extremely active system and requires a large amount of reserve energy to allow tissue maintenance at all times; thus, these gastric stem cells have always been of interest. In fact, as early as 2007, Qiao XT used galactosidase (β-galactosidase) as the driver gene for expression of the small fluorescent protein GFP through Cre recombinase technology to trace these gastric epithelial cells by lineage analysis. These experiments showed that the labeled cells are quiescent under normal physiological conditions, but under stimulation with interferon, they begin to proliferate and differentiate into a variety of gastric lineage cells of the gastric gland; thus, this population of cells can be defined as a subgroup of gastric stem cells [113]. To study gastric stem cells, Nam et al. used Mist1-CreER mice to trace the lineage of mature chief cells based on the previously proposed identity of mucoid epithelial metaplasia as a precancerous lesion of gastric cancer. The experiments showed that under conditions of gastric mucosal loss and inflammation, mature chief cells play the role of stem cells and begin to expand and transdifferentiate to promote damage repair [114]. Later, lineage tracing technology was used to show that some chief cells located in the small curvature of the stomach exhibited LGR5 transcriptional activity and did not have the ability to generate epithelial metaplasia [115]. LGR5 is a specific marker of gastric stem cells. Exploiting this characteristic, specific knockout of Smad4 and PTEN was performed in gastric stem cells. Gastric adenomas in mice rapidly progressed to invasive intestinal gastric cancer (IGC) [116]. Gastric stem cells expressing LGR5 are the initiating factors for the progression of cancer malignancy (Fig. 5c). In a recent study, Seidlitz et al. used lineage tracing technology to establish a CreERT2 mouse model with a phenotype similar to that of human gastric cancer. Then, they extracted tumor tissue from these mice and investigated the effects of chemotherapeutic drugs and various pathway inhibitors [117]. In summary, lineage tracing can be used not only to explore the presence and differentiation potential of gastric stem cells but also to study their role in disease models to support the discovery of tumor stem cell-targeted drugs for clinical application.

#### The intestine

In 2006, Garrison et al. used Cre-loxp recombinase to study the effect of hnf4-alpha on colonic epithelial development. They knocked down hnf4-alpha expression in colonic epithelial cells of mouse embryos and found that the mouse colon failed to form. The normal crypt structure and colon function were also affected [118]. However, in addition to its use in developmental studies of the gastrointestinal tract, lineage tracing technology can also be used to investigate the normal mechanisms and functions of the gastrointestinal tract. In an experiment to study gastrointestinal motility, another group specifically knocked out the MLCK gene in smooth muscle cells through the Cre-loxp recombinase system, which significantly reduced the phosphorylation level of RLC and caused severe intestinal dyskinesia and weak peristalsis [119]. Barker et al. used this upgraded system to trace crypt basal columnar cells for 60 days and found that the lgr5 gene is a marker of intestinal stem cells [120]. The intestinal epithelium has the ability to rapidly self-renew because of the support of intestinal stem cells. However, intestinal stem cells attain a steady state during loss compensation, mainly through a pattern of neutral drift [121]. Combined with the results of previous studies, this observation indicates that in the absence of Lgr5+ stem cells, cycling secretory progenitors and quiescent secretory precursors can restore the plasticity of the cells to compensate for the missing Lgr5+ stem cells [122, 123]. Tetteh et al. crossed Alpi-CreER^+/+^; R26RLacZ^+/−^ mice with lgr5dc-gfp^+/−^ mice, labeled Alpi+ cells with the fluorescent reporter tdTomato and lgr5+ cells with GFP, and performed lineage analysis on the labeled cells. tdTomato and GFP fluorescence co-localized at the base of the crypt [124]. This study confirmed that Alpi+ cells have an abundance of transcripts and that when stem cells are damaged, Alpi+ enterocytes can differentiate into Lgr5+ stem cells and maintain homeostasis of the Lgr5+ intestinal stem cell population. Snippert et al. used Lgr5-EGFP-Ires-CreERT2 mice to explore the growth environment of Lgr5+ intestinal stem cells and found that Paneth cells play an important role in this environment. CD24+ Paneth cells express specific growth factors necessary for stem cells, such as EGF, TGF inhibitors, the Notch ligand Dll4, and Wnt3 [125]. In 2019, Guiu et al. raised the question of whether LGR5+ cells are the only adult intestinal stem cells. They conducted scRNA-seq of the embryonic intestine at 16.5 days and found that the LGR5+ population is indeed an important source, although not the only source, of adult intestinal stem cells. A group of cells with differentiation potential also resides in the embryonic intestine. Researchers traced cell lines expressing KRT19 and found that these cells have a self-renewal ability equivalent to that of LGR5+ cells [126]. Thus, the intestinal tract has the function of rapid regeneration, and this important function cannot be separated from intestinal stem cells. Intestinal stem cells are an enigmatic population with a short life span and rapid renewal ability. Therefore, lineage tracing technology lays a foundation for understanding the origin and fate of intestinal stem cells.

To date, in addition to being used to study gastrointestinal tract physiology, lineage tracing approaches have also been used to simulate disease lineage models. For example, Manicassamy et al. used the CD11c promoter to drive cre expression to knock out β-catenin in intestinal immune cells and induce inflammatory bowel disease. Their study showed that Wnt/β-catenin signaling pathways in intestinal dendritic cells can regulate intestinal inflammation [127]. In 2019, Lei Zhou et al. used Ncr1cre-Il2f/f mice to study the molecular mechanism by which homeostasis is regulated in the intestine and found that in addition to a group of IL-2-expressing CD4+ T cells, a group of ILC3 cells were also an important source of IL-2 and that activation of the ILC3-IL-2 pathway is dependent on IL-1β [128].

Subsequently, Wang et al. established a Pdgfrb-Cre; Itpr1^fl/fl^ mouse model by studying the physiological function of the IP 3 R gene in the gastrointestinal tract. Gastrointestinal motility disorders were found in these mice, and IP 3R deficiency was found to have negative effects on gastrointestinal smooth muscle contractility [129]. In addition, Mariko et al. used tamoxifen to induce LGR5 gene silencing in LGR5-cre mice and later discovered that ablation of LGR5+ tumor stem cells that were isolated from these mice and xenotransplanted into other mice induced tumor regression, but the reappearance of LGR5+ tumor stem cells induced tumor regrowth (Fig. 5d). This group also combined chemotherapy administration and cell lineage studies to explore the plasticity of tumor stem cells [130, 131]. In a follow-up study on colon cancer dynamics, compilation and quantitative analysis of the lineage tracing data showed that the number of non-functional cancer stem cells increased during tumor treatment. Therefore, the experimental team studied intrinsic approaches to suppress cancer progression in-depth [132]. However, importantly, by combining innovative lineage tracing technologies, we can further explore tumor occurrence and development to study factors affecting the tumor microenvironment and elucidate the effects of exogenous drugs, both of which have an important role in the treatment of clinical diseases.

## Conclusion and future directions

Simulating human development and disease in experimental animals is a long and difficult process. From in vitro cell models to animal models to clinical experiments, our understanding of the digestive system has been gradually enhanced. The application of lineage tracing technology has filled knowledge gaps in most aspects of the digestive system. Recently, with the development of lineage tracing technology based on traditional gene-targeting technology combined with advanced methods such as genetic barcode and single-cell sequencing technology, we have obtained a more accurate understanding of organ development and disease origin and development.

This article summarizes the application of lineage tracing technology in digestive system research. By labeling the site of a single-specific cell, we can trace its fate. To date, two brilliant studies have shown that one important application of lineage tracing is the study of stem cells. Stem cells are a focus in almost all research fields and have extraordinary multi-directional differentiation potential. Therefore, researchers used lineage tracking technology to track the movement of stem cells and study the fate and function of progeny cells. Another study focused on mature differentiated cells through lineage tracing and showed that in the case of body damage, differentiated mature cells exhibit plasticity, can dedifferentiate into stem-like cells, and can even directly transdifferentiate into the lineage of the damaged cells.

In summary, lineage tracing technology is increasingly widely used. It can be applied to the developmental biology of organs. It can also be used to explore changes in the microenvironment by the establishment of disease models or even to screen drugs. By labeling specific cells and administering targeted drugs, we can evaluate the efficacy of the drugs and the prognosis of the disease. Through lineage tracing technology, we can clearly determine the efficacy of the drug and its effect on other surrounding cells.

However, the application of lineage tracing technology has limitations. For example, in the study of the stomach, lineage tracing models often use tamoxifen induction, which damage the gastric mucosa and may cause off-target effects. Moreover, we found that most pancreatic studies require the combination of multiple lineage tracing methods, especially single-cell sequencing. However, the cost of single-cell sequencing is relatively high; thus, we should explore more diversified lineage tracing methods to solve the current problems in scientific research and reveal the complex pathological and physiological mechanisms of the body, which will be further verified in the clinic.

## Data Availability

Not applicable.

## Abbreviations

HPCs: Hepatic progenitor cells
BECs: Biliary epithelial cells
HCC: Hepatocellular carcinoma
ICCA: Intrahepatic cholangiocarcinoma
DNA: Deoxyribonucleic acid
GFP: Green fluorescent protein
CDE: Choline-deficient, ethionine-supplemented
Mdm2: Mouse double minute 2 homolog
Dsh: Disheveled, a family of proteins
LRP: Low-density lipoprotein-related protein
RTK: Receptor tyrosine kinase
ROR2: Receptor-related 2
KO: Knockout
AAV: Adeno-associated virus
GEMM: Genetically engineered mouse model
Nf2: Neurofibromatosis type 2
DDC: 3,5-Diethoxycarbonyl-1,4-dihydrocollidine
TAA: Thioacetamide
MDA: Methylenedianiline
DEN: *N*-nitrosodiethylamine
BDL: Bile duct ligation
HBV: Hepatitis B virus
